# Neural Mechanism of Repeated Transcranial Magnetic Stimulation to Enhance Visual Working Memory in Elderly Individuals With Subjective Cognitive Decline

**DOI:** 10.3389/fneur.2021.665218

**Published:** 2021-07-12

**Authors:** Meng Liu, Zhi-Yu Nie, Ren-Ren Li, Wei Zhang, Li-He Huang, Jie-Qun Wang, Wei-Xin Xiao, Jialin C. Zheng, Yun-Xia Li

**Affiliations:** ^1^Department of Neurology, Tongji University School of Medicine, Tongji Hospital, Tongji University, Shanghai, China; ^2^Research Center for Ageing Language and Care, School of Foreign Languages, Tongji University, Shanghai, China; ^3^School of Medicine, Tongji University, Shanghai, China

**Keywords:** repetitive transcranial magnetic stimulation, visual working memory, N2PC, contralateral delayed activity, β oscillations

## Abstract

Visual working memory (VWM), the core process inherent to many advanced cognitive processes, deteriorates with age. Elderly individuals usually experience defects in the processing of VWM. The dorsolateral prefrontal cortex is a key structure for the top-down control of working memory processes. Many studies have shown that repeated transcranial magnetic stimulation (rTMS) improves VWM by modulating the excitability of neurons in the target cortical region, though the underlying neural mechanism has not been clarified. Therefore, this study sought to assess the characteristics of brain memory function post-rTMS targeting the left dorsolateral prefrontal cortex. The study stimulated the left dorsolateral prefrontal cortex in elderly individuals by performing a high-frequency rTMS protocol and evaluated behavioral performance using cognitive tasks and a VWM task. Based on the simultaneously recorded electroencephalogram signals, event-related potential and event-related spectral perturbation analysis techniques were used to investigate the variation characteristics of event-related potential components' (N2PC and CDA) amplitudes and neural oscillations in elderly individuals to elucidate the effect of high-frequency rTMS. The results found that rTMS enhanced VWM performance and significantly improved attention and executive function in elderly individuals with subjective cognitive decline. We therefore speculate that rTMS enhances VWM by increasing the N2PC and CDA amplitude, alongside increasing β oscillation activity. This would improve the attention and allocation of resources in elderly individuals such as to improve an individual's VWM.

## Introduction

With the advent of an aging society, the percentage of people with Alzheimer's disease (AD) and dementia increases with age, affecting 3% of people aged 65–74 years, 17% of people aged 75–84 years, and 32% of people aged 85 years and older (1). The main clinical manifestations consist of cognitive dysfunction, memory loss, and abnormal changes in personality, which seriously threaten human health and life quality. Dementia poses a high burden on society and health care, both in terms of the suffering inflicted on patients and care givers and in financial costs (2). Subjective cognitive decline (SCD) is considered a risk factor for AD (3). SCD might be an important “time course” for early AD intervention (4). However, there are no effective drugs that reverse or delay the onset of AD (5). As a series of drug clinical trials carried out both in China and abroad have failed to generate effective results (6), more researchers have focused on non-pharmacologic interventions. Non-pharmacologic interventions are better accepted than pharmacologic interventions for patients with mild clinical symptoms of SCD.

Repetitive transcranial magnetic stimulation (rTMS) is believed to modulate brain function through methods of electromagnetic induction (7). As rTMS treatment is non-invasive, highly targeted, with certain effects in enhancing cognitive function (8), and both safe and reliable (9), it has been used in many studies to improve human memory function and has yielded good results as a non-pharmacological treatment (10). A previous study on the effect of rTMS on improving memory was based on young people, and only a few studies have shown that rTMS enhances memory function in elderly individuals (11). However, most studies lack further discussions on the neural processing mechanisms of memory.

Visual working memory (VWM) represents the temporary storage and processing of visual information acquired from the environment for subsequent cognitive processing and serves as the basis for advanced cognitive processes (12–14). VWM declines over the course of healthy aging (15), and studies have found defects in the storage and processing of VWM in the brain at this stage (16–18). Evidence from event-related potential (ERP) studies shows that for components related to visual working memory, such as contralateral delayed activity (CDA) and N2PC, their reduction by age is more pronounced in older individuals (19). The CDA reflects the encoding and maintenance of item representations in visual working memory. The amplitude of this CDA increases significantly as the number of representations being held in memory increases (20). The N2PC component is related to spatial selective attention, which reflects the spatial selective processing of the current task (21).

Studies have shown that activating the left dorsolateral prefrontal cortex (DLPFC) contributes to the filtering of distractors and plays an important role in the storage and processing of VWM (22). However, it remains unclear how rTMS improves VWM by activating DLPFC and which stage of information processing of VWM is affected by rTMS. Moreover, related cortical electrical activities require further exploration. Targeting the neural mechanisms underlying VWM improvement will help us further explore the mechanisms linked to TMS.

Therefore, this study sought to enroll elderly individuals aged ≥50 years with SCD to observe the characteristics of brain memory function post-stimulus after targeting the left DLPFC with rTMS. Based on the change in amplitudes of the event-related potential (ERP) components [N2PC and contralateral delayed activity (CDA)], as well as the neural oscillation activity in each frequency band (θ, α, β, γ), during the neural processing related to a VWM task, we sought to assess the effects of rTMS on improving brain memory function and the neural mechanisms by which rTMS enhances the VWM of elderly individuals with SCD.

## Materials and Methods

### Participants

The Department of Neurology of Tongji Hospital enrolled participants with memory decline as their primary complaint. We included 25 elderly patients with SCD (14 males and 11 females, mean age 69.92 ± 1.93 years) based on the inclusion and exclusion criteria. The Ethics Committee of the Tongji Hospital approved the study [No. (Tongji hospital in Shanghai, China) ethical review 429] on 10 Jan 2019, and all participants provided written informed consent.

#### Inclusion Criteria

1. Age ≥ 50 years (no gender limitation)
2. Primary school education or above
3. SCD-Q9 screening questionnaire score > 5
4. Global cognitive function and daily life ability in good condition
5. Normal objective memory assessment
6. No other mental and neurological diseases

#### Exclusion Criteria

1. Patients with diagnosed dementia and a history of cerebrovascular disease.
2. Patients with a consciousness disorder, severely impaired vision, hearing, aphasia disorder, and other physical diseases that seriously affect neuropsychological testing.
3. Patients with severe primary diseases of the liver, kidney, hematopoietic and endocrine systems; mental illness; epilepsy; Parkinson's disease; diabetes; heavy drinking; drug abuse; and malignant tumors.
4. Patients with rTMS contraindications with metal implantable devices, including metal clips, plates, or rods; stents and filters; implanted electrodes for deep brain procedures such as vagal stimulation and electroconvulsive therapy; pacemakers and medication pumps; hearing implants; bullets or metal fragments.

### Experimental Protocol

All participants were required to provide written informed consent and underwent related examinations before participating in the experiment. Cognitive screening with an SCD survey of nine items; examinations including cranial computed tomography or magnetic resonance imaging (MRI); and blood biochemical assessments of folic acid, vitamin B12, thyroid function (free triiodothyronine, free tetraiodothyronine, and thyroid stimulating hormone), treponema pallidum, and HIV antibodies were conducted. All participants were required to complete the Neuropsychological Test Battery, which consisted of Global cognitive function [Mini-Mental State Examination (MMSE)], memory (Hopkins verbal learning test, Logical memory test, Wechsler memory scale), executive function [Shape trails test (STT)], language (Boston naming test, Verbal fluency test-vegetables), and visuospatial function assessments (Rey-Osterrieth Complex Fig.) as well as attention (Digit span) and instrumental evaluation of daily living abilities. All participants were required to undergo Digit Symbol Coding test (DSCT), Stroop test (word-Stroop test, color-Stroop test, word-color Stroop test), and Digit span test, including Digit Span Forward test (DSFT) and Digit Span Backward test (DSBT) pre-stimulation and post- stimulation. These were completed in a neuropsychological assessment room that was quiet without interruption, and participants were instructed to remain relaxed. The neuropsychological assessors were unaware of the treatment plans of the participants. The flow chart of the experiment can be seen in Supplementary Material.

#### rTMS Procedure

rTMS was performed with an active figure-8 coil (MC-B70, DK-3520, Denmark) and a Mag Pro X100 stimulator with Mag Option (Mag Pro R30, Denmark). Resting motor threshold (RMT) was then defined as the rTMS pulse intensity producing, on average, a motor evoked potential (MEP) of a 50 μV peak-to-peak amplitude. MEP referred to the contraction potential recorded at the corresponding target tendon after rTMS stimulated the cerebral motor cortex. The measurement method was as follows: participants were seated upright and remained relaxed on a chair with their hands spread flat on the legs. The coil's center was placed tangentially above the patient's left motor cortex to trigger a single pulse stimulation. We observed the patient's right hand, and we would lower the set threshold until the patient's right hand stopped the maximum threshold for automatic movement if any involuntary movement occurred. The stimulation site was the left DLPFC, determined according to the Beam F3 system (23). All participants were given 10 s trains of 10 Hz rTMS at 100% of RMT for a total of 1,500 pulses with an intertrain interval of 10 s.

#### Visual Working Memory Task

**Figure 1:**
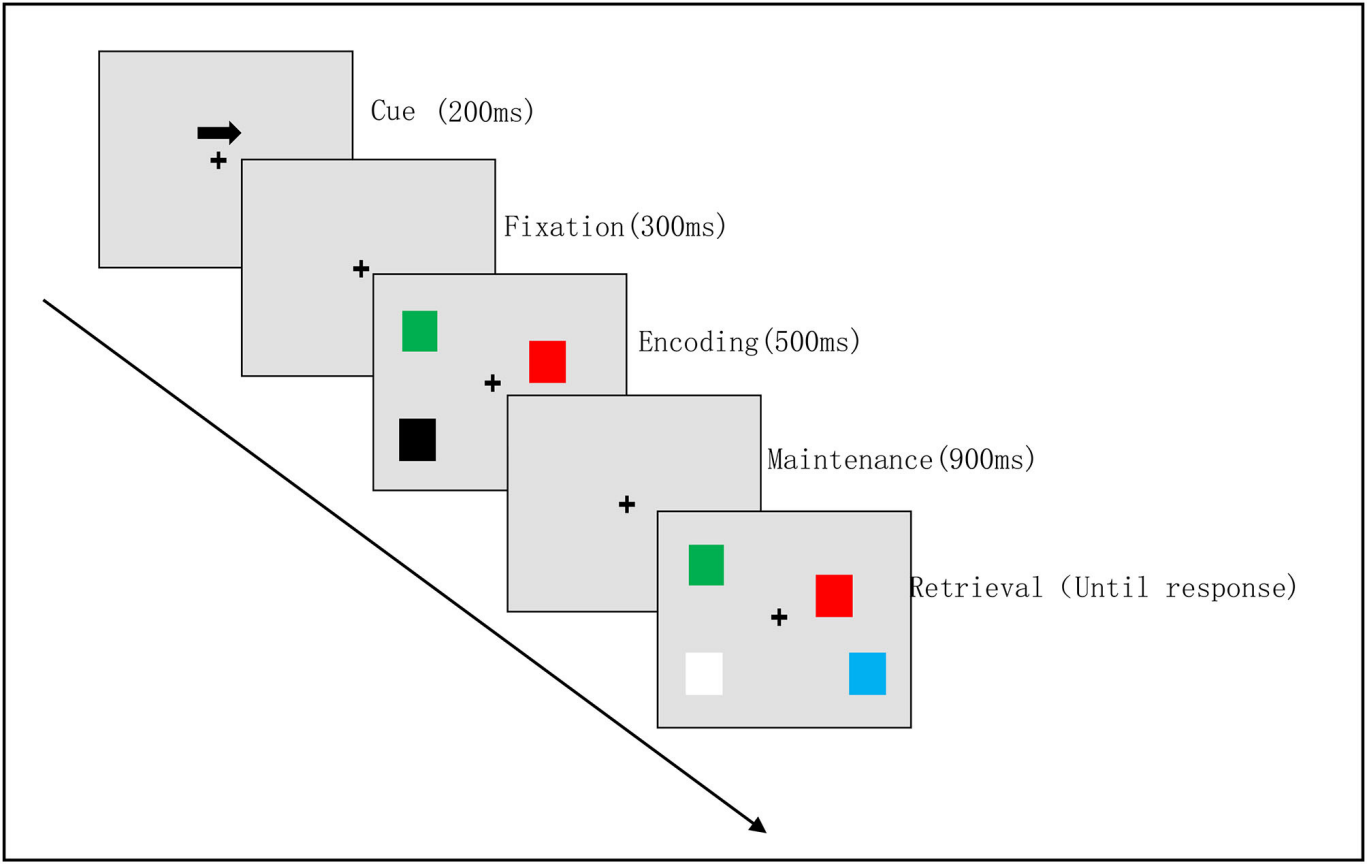
Visual working memory task procedure. Example of a visual memory trial for the right hemifield. The attention phase [cue (200 ms) with a fixation (300 ms)]; the memory encoding phase (500 ms); the maintenance phase (900 ms); the retrieval phase (until response).

The assessment of the VWM task was conducted in a quiet clinical environment, and each participant sat about 60 cm away from the computer screen. We used the Eprime software (3.0) for the improved classical VWM task in this study (24). At each trial, the cue was presented for 200 ms, followed by a blank delay for 300 ms. The memory array was presented for 500 ms, followed by a retention interval (during which only the fixation cross was presented) of 900 ms, and then the test array was presented (see Figure 1). The test array remained visible until a response was emitted. Participants were required to continuously stare at the gaze point while assessing their periphery to pay attention to the arrow's direction. The same number of squares of different colors appeared on both sides of the gaze point. After a delay of 900 ms, participants were presented with a visual search array as their next response. The same color blocks as used in the sample sequence appeared on both sides of the fixation point. Participants were required to assess the color of the square on the screen on the side of the arrow pointing to the opposite direction. If the color of the second set of blocks was different from that of the first set of blocks, participants pressed the right button; if both sets of blocks were identical, participants pressed the left button. The VWM task consisted of two blocks of 100 trials each.

#### Electroencephalogram Processing and ERP Analysis

Electroencephalogram (EEG) recordings were conducted in a quiet environment. A Neuroscan Acquire software using a Synamps 2 amplifier (Compumedics, Melbourne Australia) with 64 single Ag/AgCl scalp electrodes were used to record the participants' EEG activity. Four facial electrodes were positioned adjacent to the left and right outer canthus of each eye and above and below the left orbit to measure eye movement. The data were processed in MATLAB (The MathWorks Inc., Natick, MA) using EEGLAB toolbox (25) and custom codes. Electrodes were grounded to Ground (GND) and referenced online to an electrode between the CZ and CPZ electrode positions. Impedances were kept below 10 KΩ before recording. The EEG was sampled at 1,000 Hz with an online bandpass of 0.01–250 Hz. Next, we recorded the participant's EEGs movement continuously for more than 5 min and used an amplitude threshold of 200 μV to eliminate artifacts (blinks and saccades). The EEG was segmented into 1,700 ms epochs from 200 ms before the memory array onset to 2,000 ms after the memory array onset. All channels except for the HEOG and VEOG channels required an amplitude threshold of 120 μV to exclude bad blocks containing other artifacts, such as those induced by head movement or muscle activity.

The mean amplitude difference at the PO7/8 electrode was calculated by subtracting the mean activity on the opposite side. ERP difference waves were computed by subtracting the ERP waveforms obtained from electrodes located on the hemisphere ipsilateral to the target from the ERP waveforms acquired from symmetrical electrodes on the contralateral hemisphere. The contralateral waveform for the target was the average of the waveform from the left hemisphere electrode when the target was in the right visual field and the waveform from the right hemisphere electrode when the target was in the left visual field. Similarly, the ipsilateral waveform for the target was the average of the waveform from the left hemisphere electrode when the target was in the left visual field and the waveform from the right hemisphere electrode when the target was in the right visual field. The statistical analysis focused on the two ERP components: N2PC (170–290 ms) and CDA (400–600 ms). For N2PC, in the time window from 170 to 290 ms after stimulation, the most significant negative peaks on different waveforms were extracted pre-stimulation and post-stimulation. As for the CDA, we extracted the time windows' mean amplitude from 400 to 600 ms after stimulation.

#### Time–Frequency Analysis

Time–frequency distributions (TFDs) of the EEG time course was obtained using a windowed fast Fourier transform with a fixed 300-ms Hanning window. For each estimated frequency, TFDs were baseline corrected using the frequency from the pre-stimulus interval (300–500 ms), according to the formula: $ER\left(t,f\right)=\frac{\left[F\left(t,f\right)2R\left(f\right)\right]}{R\left(f\right)},\;$where F(t, f) is the signal power at a given time t and at a given frequency f, and R(f) is the signal power of the frequency f averaged within the reference interval (26). For each participant, grand average TFDs were computed for pre-stimulus and post-stimulus conditions. We calculated the event-related spectral perturbation (ERSP) value by dividing the respective mean baseline spectra from each of the individual response epoch's spectral transforms. We estimated spectral power at a frequency range of 1–40 Hz, in 0.5 Hz steps; for each time course, a complex time–frequency estimate F (t, f) at each point (t, f) of the time–frequency plane, extending from 300 to 200 ms (in steps of 1 ms) in the time domain was assessed. The average ERSP value for each participant and each electrode was calculated separately. For all brain regions, the difference in ERSP values for each frequency band (θ, α, β, γ) at all time points under the two conditions (pre- and post-stimulus) was calculated. The spectrum power value was calculated by averaging the absolute power obtained by ERSP analysis of different frequency bands. The frequency bands of interest were the θ (4–7 Hz), α (8–12 Hz), β (13–30 Hz), and γ (30–40 Hz) bands.

#### Statistical Analysis

Statistical analyses were carried out in SPSS (Version 20.0, Tongji University, China). Data were expressed as mean ± standard deviation values or percentages, and the demographics were described. The VWM behavioral performance as well as other related cognitive function test results, including digit span test scores, DSCT scores, and Stroop test scores, were assessed with a paired *t*-test. Electrophysiological findings consisting of the N2PC and CDA amplitudes and spectrum power of region of interest were compared with a paired *t*-test. A value of two-sided *p* < 0.05 was considered statistically significant.

## Results

### Baseline Participant Characteristics

Table 1 represents the demographic data of participants involved in the current study, which included: age; sex; educational years; the total MMSE score; the total Hamilton Depression Scale - 17 items (HAMD-17) score; scores of Hopkins verbal learning, immediate recall, 5-min delayed recall, and 20-min delayed recall tests; Boston naming score and verbal fluency score; and scores of shape trails test (STT_A and STT_B) and Rey-Osterrieth Complex Fig. scores (copy and recall).

**Table 1:** Demographic data.

	**Subjects (*n* = 25)**
Age (years)	69.92 ± 1.93
Gender (M/F)	14/11
**Handedness (left/right/both)**	
Education (years)	11.40 ± 0.66
MMSE	26.24 ± 0.57
HAMA	5.72 ± 0.79
HAMD	4.32 ± 0.83
**Memory function domain**	
Hopkins verbal learning test (HVLT) Immediate recall	19.68 ± 1.09
HVLT - Delayed recall (5 min)	5.8 ± 0.78
HVLT - Delayed recall (20 min)	6 ± 0.68
Logical memory test (Wechsler memory scale)	8.00 ± 0.59
**Language function domain**	
Boston naming test	23.20 ± 0.79
Verbal fluency test-vegetables	13.48 ± 0.76
**Executive function domain**	
Shape trails test (STT-A)	58.28 ± 5.00
Shape trails test (STT-B)	150.44 ± 1.95
Rey-Osterrieth Complex Figure Test (The copy scores)	31.96 ± 1.07
Rey-Osterrieth Complex Figure Test (The recall scores)	13.16 ± 1.82

*MMSE, Mini-Mental State Examination; HAMA, Hamilton Anxiety Scale; HAMD, Hamilton Depression Scale*.

### Behavioral Performance

**Figure 2:**
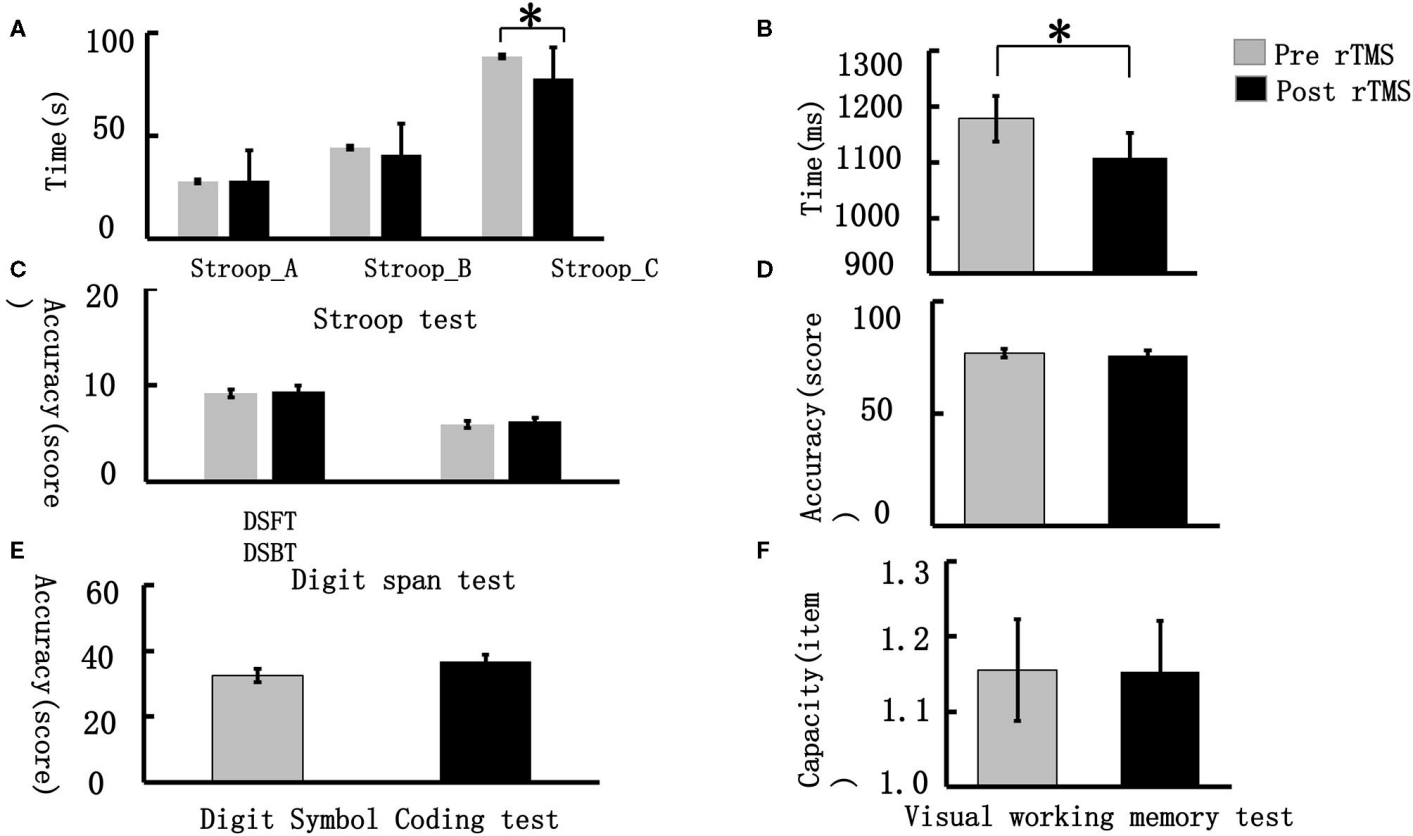
Visual working memory behavioral performance. **(A)** represents the performance difference between pre- and post-stimulus in the word-Stoop, the color-Stroop and word-color Stroop tests (from left to right); **(B)** demonstrates the reaction time difference during the VWM task between the pre- and post-stimulus periods; **(C)** illustrates the performance differences between the pre- and post-stimulus during the Digit Span Forward test (DSFT) and Digit Span Backward test (DSBT); **(D)** demonstrates the accuracy differences during the VWM task between the pre- and post-stimulus periods; **(E)** represents the performance differences between the pre- and post-stimulus periods in the Digit Symbol Coding test; **(F)** demonstrates the capacity difference during the VWM task between the pre- and post-stimulus periods. *p < 0.05, **p < 0.01.

We compared the differences in VWM performance pre- and post-stimulus in terms of accuracy, reaction time, and memory capacity. The results showed that the single rTMS significantly decreased the reaction time during the VWM task (pre-stimulus vs. post-stimulus: 1,177.92 ± 202.62 vs. 1,108.22 ± 217.79, *t* = 2.577, *p* = 0.017). In addition, we compared the performance difference in the DSCT, Digit Span, and Stroop tests. The participants showed significant improvement in the DSCT performance post-stimulus (pre-stimulus vs. post-stimulus: 32.56 ± 10.28 vs. 36.84 ± 10.28, *t* = −3.815, *p* = 0.001). For the Stroop test, the participants took less time post-stimulus to complete this task compared with that taken pre-stimulus (pre-stimulus vs. post-stimulus: 88.32 ± 25.61 vs. 77.84 ± 19.32, *t* = 3.25, *p* = 0.003). However, no significant difference was observed in the performance of the Digit Span test (see Figure 2).

### ERPs

#### N2PC

**Figure 3:**
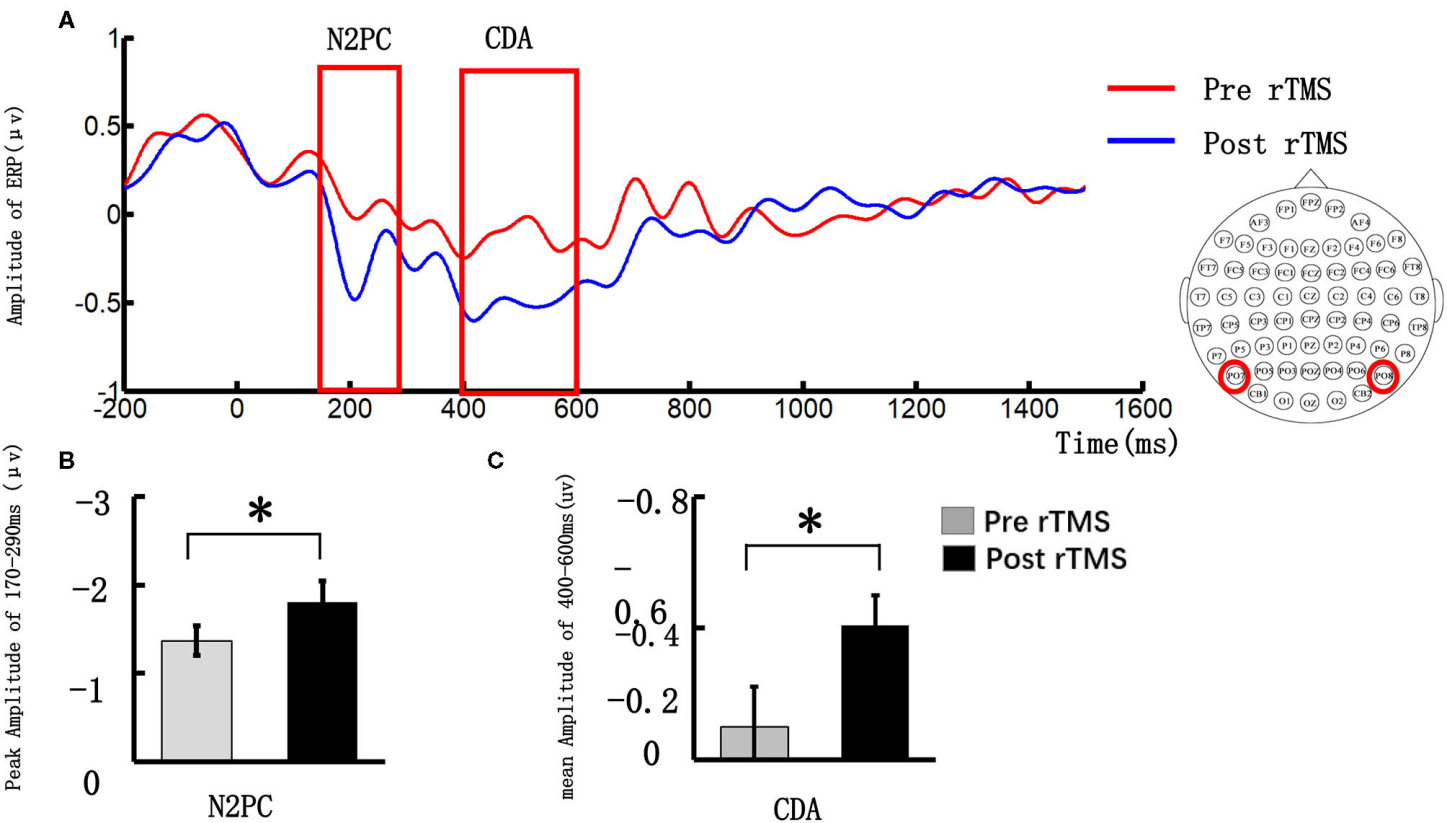
Grand average event-related potentials and amplitude differences of the N2PC and CDA pre- and post-stimulus repeated transcranial magnetic stimulation (rTMS). **(A)** illustrates the event-related potential (ERP) amplitude in the visual memory trial; the N2PC time window (170–290 ms) and CDA time window (400–600 ms) were highlighted in the red strip; **(B)** demonstrates the N2PC amplitude difference during the VWM task between the pre- and post-stimulus periods; **(C)** demonstrates the CDA amplitude difference during the VWM task between the pre- and post-stimulus periods. *p < 0.05.

Figure 3A illustrates the event-related potential (ERP) amplitude in the visual memory trial. We extracted the peak value during the 170–290 ms time window and performed statistical tests. As Figure 3B shows, the post-stimulus N2PC amplitude was significantly larger than that obtained pre-stimulus (pre-stimulus vs. post-stimulus: −1.37 ± 0.84 vs. −1.81 ± 1.16, *t* = 2.27, *p* = 0.049).

#### CDA

We extracted the mean value in the 400–600 ms time window and performed a statistical analysis. Figure 3C illustrates that the CDA amplitude was significantly larger post-stimulus than pre-stimulus (pre-stimulus vs. post-stimulus: −0.12 ± 0.76 vs. −0.51 ± 0.58, *t* = 2.27, *p* = 0.032).

#### Time–Frequency Distributions With Baseline Correction

**Figure 4:**
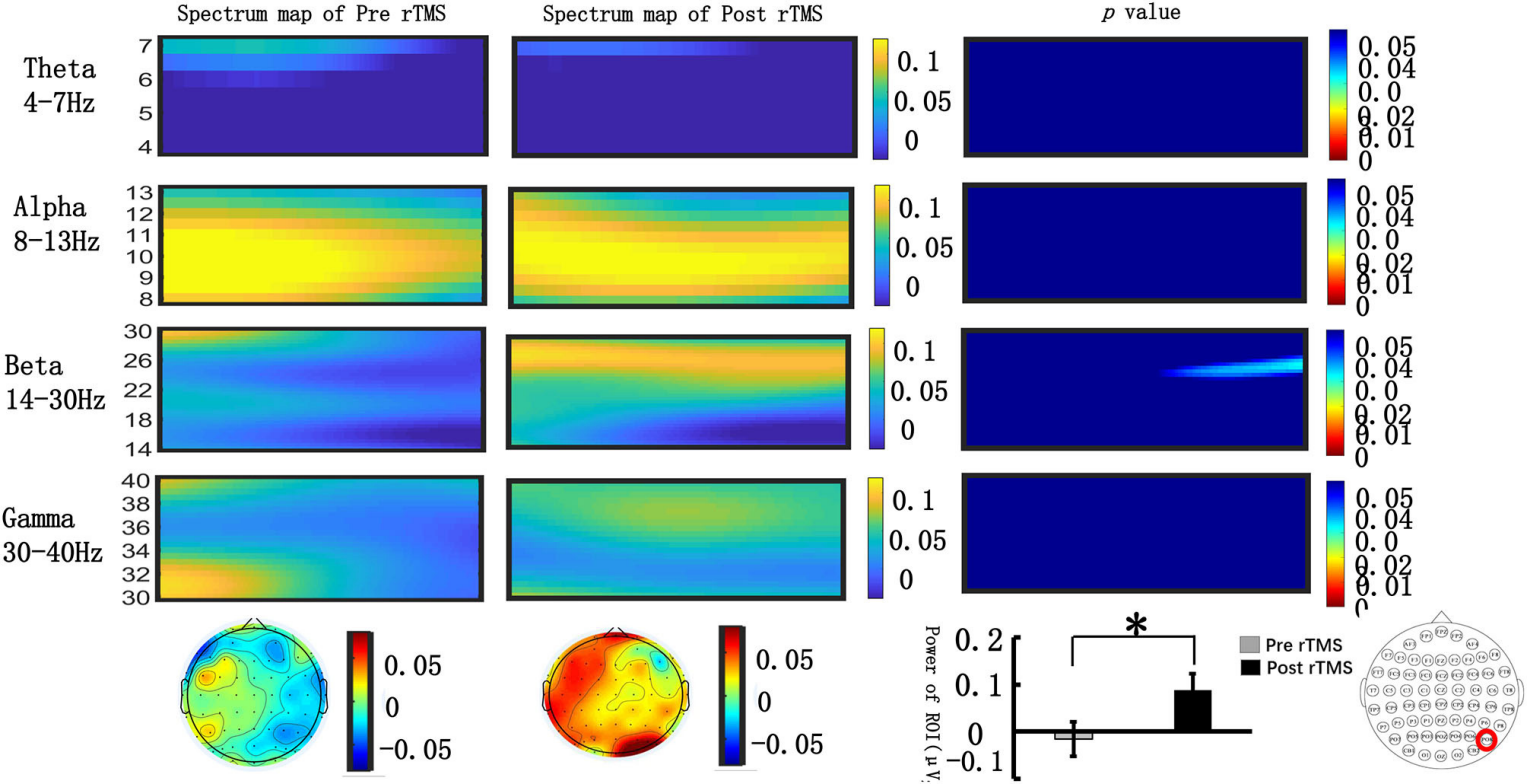
Grand average time–frequency distribution (TFD) and β oscillation scalp topographies over the occipital lobe during the N2PC time window pre- and post-repeated transcranial magnetic stimulation (rTMS). The time–frequency distribution over the occipital lobe (PO8) in the θ (4–7 Hz), α (8–13 Hz), β (13–30 Hz), and γ bands (30–40 Hz) during the N2PC time window (170–290 ms); the spectrum map of pre- and post-rTMS are listed separately in the first and second line. The third line represents the *p*-value of every frequency point and time point of spectrum power pre- and post-rTMS. Significant differences were found in the β (24–28 Hz) oscillation scalp topographies over the occipital lobe during the N2PC time window (268–290 ms); bar graph differences between pre- and post- rTMS time periods are shown at the bottom of figure. *p < 0.05.

**Figure 5:**
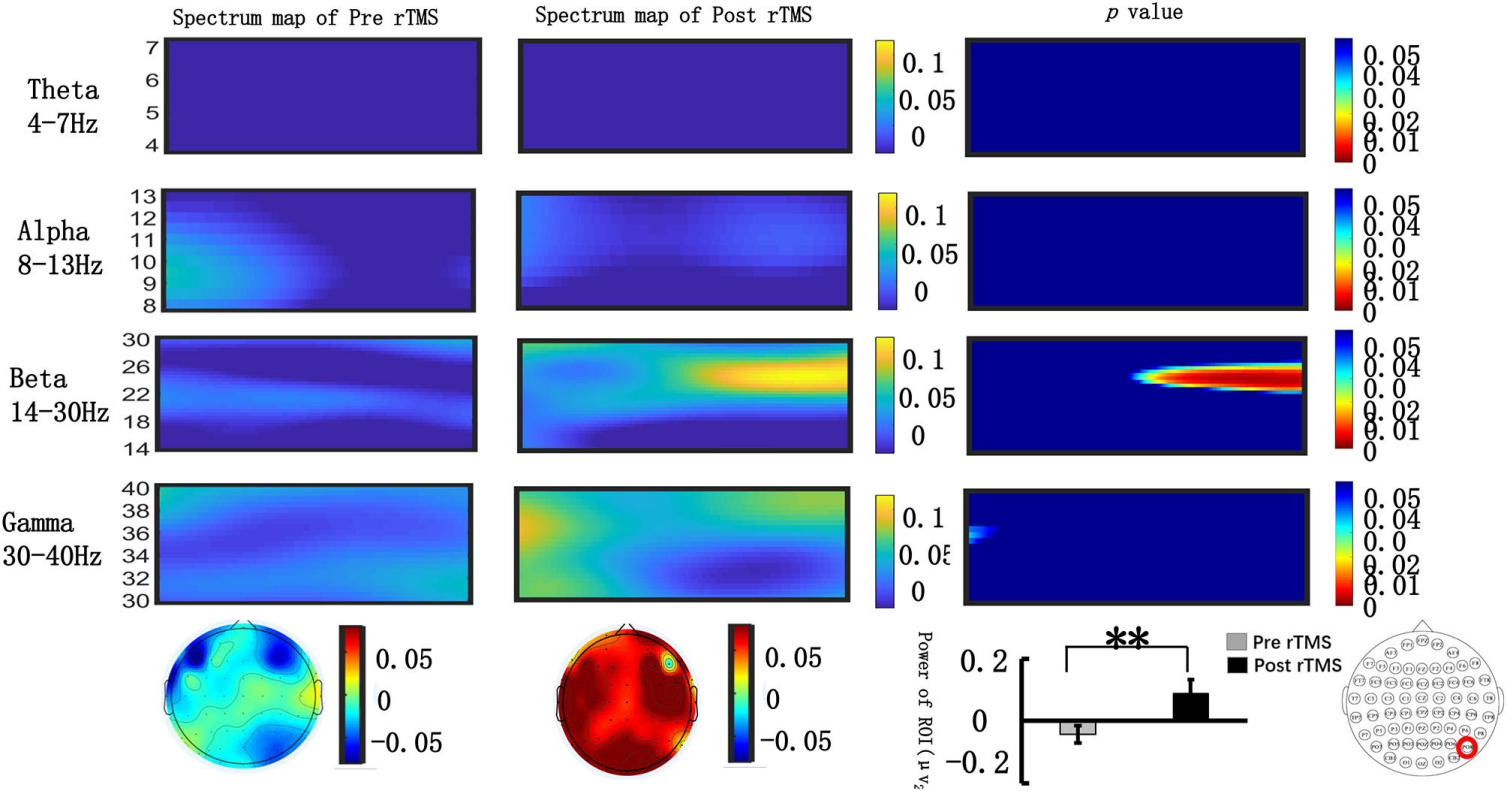
Grand average time–frequency distribution (TFD) and β oscillation scalp topographies over the occipital lobe during the N2PC time window pre- and post-repeated transcranial magnetic stimulation (rTMS). The time–frequency distribution over the occipital lobe (PO8) in the θ (4–7 Hz), α (8–13 Hz), β (13–30 Hz), and γ bands (30–40 Hz) during the CDA time window (400–600 ms); the spectrum maps pre- and post-sTMS are listed separately in the first and second line. The third line represents the *p*-value of every frequency point and time point of spectrum power pre- and post-sTMS. Significant differences were found in the β (22–26 Hz) oscillation scalp topographies over the occipital lobe during the CDA time window (510–600 ms); bar graph differences of the pre- and post-time periods are shown at the bottom of figure. **p < 0.01.

We extracted the spectral power of all frequency bands (θ, α, β, γ) in the occipital lobe during the N2PC (170–290 ms) and CDA (400–600 ms) time windows and performed a paired *t*-test. Figure 4 shows that during the N2PC time window (268–290 ms), the β band (24–28 Hz) spectral power in the occipital lobe significantly increased after the stimulus compared with that before the stimulus (pre-stimulus vs. post-stimulus: −0.015 ± 0.181 vs. −0.089 ± 0.178, *t* = −2.182, *p* = 0.039). During the CDA time window (510–600 ms), the β band (22–26 Hz) demonstrated a statistically significant difference in the spectrum power value in the occipital lobe post-stimulus (pre-stimulus vs. post-stimulus: −0.042 ± 0.13 vs. −0.089 ± 0.22, *t* = −2.99, *p* = 0.006) (see Figure 5). However, there were no significant differences in other frequency bands and time points.

## Discussion

In our current study, we assessed the effects of rTMS targeting the DLPFC to enhance cognitive function, especially the VWM, of elderly individuals with SCD. We monitored the characteristics of EEG signals related to VWM tasks aiming to understand the electrophysiological mechanisms underlying VWM processing. We found that rTMS improved VWM, attention, and executive function. More importantly, rTMS also enhanced the encoding and maintenance of memory according to the findings of scalp EEG signals during the VWM process.

### Cognitive Function

#### Visual Working Memory Task

We identified that a single rTMS application enhanced VWM. The behavioral findings indicated that targeting the DLPFC in the left brain improved performance in the VWM task mainly by shortening the reaction time, which was related to the speed of information processing. Therefore, our findings suggested that rTMS helped improve the information processing speed of elderly individuals with SCD, which was consistent with the findings of a previous study in young people (27). To the best of our knowledge, these results served as the first proof that a single rTMS targeting the left DLPFC improved VWM ability in this population.

However, our findings indicated that rTMS did not significantly improve the memory capacity of the VMW task, which implies that rTMS improved the information processing speed rather than the memory storage ability of the elderly. The study results also suggested that rTMS did not significantly improve the accuracy of the VMW task. Some researchers believe that there is a ceiling effect in accuracy and that reaction time can have a more meaningful variation in the working memory task (28). Variations in reaction time may be more sensitive than accuracy; due to the single session, rTMS stimulation does not significantly improve accuracy. The absence of significant difference in accuracy among the participants with SCD may be related to the ceiling effect of accuracy.

#### Other Related Cognitive Functions

We also found that the accuracy of the DSCT significantly improved after rTMS for elderly individuals with SCD. Previous studies suggested that the DSCT was closely related to VWM, and both tasks were involved in the speed of information processing (29). Our findings suggested that rTMS improved both reaction time in the VWM task and accuracy of the DSCT to some extent, which may be related to the improvement of the information processing speed of the brain.

The behavioral performance indicated that high-frequency rTMS reduced the total time taken by the participants to complete a Stroop_C task with an interference effect. However, no significant difference was seen in the total time taken for Stroop_A and Stroop_B tasks. Stroop_A test referred to the word-Stroop test, Stroop_B referred to the color-Stroop test, while Stroop_C referred to the word-color Stroop test, which assesses the ability to inhibit cognitive interference occurring when the processing of a stimulus feature affects the simultaneous processing of another attribute of the same stimulus (30). We speculated that the reaction time reduction found for incongruent trials might be associated with enhancement in inhibitory control post HF-rTMS (31). Previously published studies have also documented its application to measure other cognitive functions, such as attention, processing speed, and cognitive flexibility (32). In addition, previous studies have found that a single rTMS application reduced the response time of healthy participants in the Stroop task (33, 34); our findings suggest that rTMS improved the ability of elderly individuals with SCD to suppress irrelevant information and improved their executive function. VMW is an important component of executive function, and we found a synergistic improvement in VMW and Stroop_C task performance.

### VWM Task-Related ERP

However, behavioral measures reflected only the results of the processing and not the details of VWM processing and its underlying neural mechanisms. The processing of VMW can be divided into different phases, including memory coding, memory retention, and memory output. We compared the N2PC and CDA components, which reflect the memory coding and retention performance during VWM processing. The results showed that the amplitude of both N2PC (264–290 ms) and CDA (510–600 ms) components in the left DLPFC were significantly increased post-stimulus compared with their pre-stimulus values.

In the VWM task, N2PC is usually observed about 200–300 ms post-stimulus (35, 36), and it is an early memory component associated with attentive target selection (21). Previous studies (37, 38) confirmed that the amplitude of N2PC is related to the allocation of attention resources in visual memory tasks. The more the VMW task focused on the visual memory task, the greater the allocation of attention resources, the greater the N2PC amplitude, and the faster the task was completed (39). In addition, our study findings showed that high-frequency rTMS significantly increased the response speed of elderly individuals with SCD, and the amplitude of N2PC also increased significantly after stimulation, which may be related to the fact that after stimulation of the DLPFC, more attentional resources were allocated from top to bottom, accelerating the speed of response selection.

The CDA is a neuroelectric activity related to VWM coding and maintenance of visual information in the brain (24, 40) and emerges around 400 ms post-stimulus onset (41). Similar to N2PC, CDA is modulated by target numerosity and increases as the number of targets increases to a maximum of three/four targets (42). Studies have confirmed that individual differences in CDA reflected both the ability to maintain a distinct number of memory items and attentional control ability (43). Our behavioral findings did not show any improvement in memory capacity with rTMS, but there was a significant increase in the amplitude of CDA. Compared with behavioral performance, electrophysiological indices were more sensitive in capturing the electrophysiological characteristics of the brain during VWM processing in elderly individuals.

Our ERP findings confirmed that the amplitudes of N2PC and CDA can still be modulated by the top-down control by rTMS in elderly individuals with SCD. By increasing the amplitudes of N2PC and CDA, the allocation of attentional resources and attentional control ability are enhanced, which improves the VWM of these individuals. We speculate that their enhanced VWM storage capacity may related to the improved allocation of attentional resources and attention control capacities.

### VWM Task-Related Neural Oscillations

The ERP components (N2PC and CDA) reflect neural information processing according to the amplitude change of the scalp EEG signals, which are a comprehensive reflection of the activities of crowd neurons. During this process, neurons in the brain oscillate at different frequency bands. Neural oscillations in different frequency bands are fundamental to the maintenance of VWM and are associated with different cognitive processes involved in VWM (44). Activities in β (13–30 Hz) and γ (40 Hz) bands are considered to play vital roles in encoding, retrieving, and maintaining stimulus materials (45, 46).

We were interested in the role of neural oscillations in different stages of VWM tasks, especially in stages of presenting memory stimuli [such as N2PC (170–290 ms)] and maintaining stimuli [such as CDA (400–600 ms)]. We also tried to assess how high-frequency rTMS affects neural oscillations in various frequency bands. We employed a fast Fourier transform analysis technology to extract the ERSP values of the contralateral side [occipital lobe (PO8)] in different frequency bands (θ, α, β, γ).

In a previous VWM EEG study, β-band EEG activity was found to be related to attentional modulation (47), and β-band spectral power in the occipital lobe was correlated with visual attention; β-band power in EEG signals recorded over occipital lobe was increased compared with the pre-stimulus power. However, this phenomenon was not observed when giving a wrong response (48). Some studies also found that there was a positive correlation between the accuracy of the VMW task and the increase in the β-band power (49, 50).

We found that in the N2PC time window (264–290 ms), the ERSP value in the β (24–28 Hz) band increased significantly compared with the pre-stimulation value, and a similar effect was noted in the CDA component. These findings indicated that rTMS increased the β activity in the occipital lobe during the N2PC time window (264–290 ms) and CDA time window (510–600 ms). An increase in β activity power may be the target of rTMS intervention.

### Limitations

To our knowledge, this is the first study to focus on the effects of high-frequency rTMS (10 Hz) applied over the left DLPFC on VWM of elderly individuals with SCD. However, the current study still has certain limitations. First, the generalizability of the current findings may be limited by our use of a relatively small sample of elderly participants. Therefore, additional studies with larger samples are warranted. Second, the study used a conventional electrode cap (F3) to target the left DLPFC; this method is less accurate compared with the MRI-guided neuro navigation that targets a desired cortical region directly (51). However, many previous studies have used this technique, and the outcomes were robust and reproducible (52). Third, our research focused on the immediate effects of rTMS. However, the long-term effects and the duration of the positive effects of rTMS are still uncertain, and further research is needed in the future. Finally, our current study targeted left DLPFC, but the obvious neural electrophysiological activities and neural oscillatory activities were observed in the occipital lobe. We speculated that there may be some correlation between the electrophysiological activities in the frontal-occipital lobe. However, our study did not conduct a brain function connections analysis of different brain regions and could not clarify the characteristics of brain network changes between the frontal and occipital lobes. In future research, we will further expand the sample size and clarify the characteristics of static and dynamic brain network changes after DLPFC stimulation to further strengthen the argument.

## Conclusions

Our research revealed that high-frequency rTMS significantly improved the reaction time, attention, ability to suppress irrelevant information, and executive function of elderly individuals with SCD when completing VWM tasks. The findings of electrophysiological studies based on ERP suggested that rTMS increased the amplitudes of N2PC and CDA, which reflect the memory coding and maintenance stages of VWM processing, in the occipital lobe. Additionally, β oscillation activity was enhanced during the N2PC and CDA time windows. We speculated that high-frequency rTMS improved the VWM ability of elderly individuals by activating the left DLPFC and modulating top-down control, aiming to increase the amplitudes of N2PC and CDA, as well as the β oscillation activity, in the occipital lobe. Our study confirmed the potential therapeutic targets for enhancing VWM in SCD to lay forth a scientific theoretical basis for future clinical applications.

## Data Availability Statement

The raw data supporting the conclusions of this article will be made available by the authors, without undue reservation.

## Ethics Statement

The studies involving human participants were reviewed and approved by The Ethics Committee Shanghai Tongji Hospital. The patients/participants provided their written informed consent to participate in this study.

## Author Contributions

ML: study design, data collection, statistical analysis, data interpretation, manuscript preparation, and literature search. Z-YN: study design, data interpretation, manuscript revision, reviewing, and editing. R-RL: data collection, statistical analysis, and literature search. WZ, J-QW, and W-XX: data collection and literature search. L-HH: manuscript revision, interpretation, reviewing, and editing. J-LZ: study design, supervision, manuscript revision, reviewing, and editing. Y-XL: study design, supervision, manuscript revision, funding acquisition, and writing (reviewing and editing). All authors contributed to the article and approved the submitted version.

## Conflict of Interest

The authors declare that the research was conducted in the absence of any commercial or financial relationships that could be construed as a potential conflict of interest.
