# Supplementary figures and images for: Neural Mechanism of Repeated Transcranial Magnetic Stimulation to Enhance Visual Working Memory in Elderly Individuals With Subjective Cognitive Decline
Source: Front Neurol. 2021 Jul 12;12:665218. doi: 10.3389/fneur.2021.665218 (PMC8320844; doi:10.3389/fneur.2021.665218)

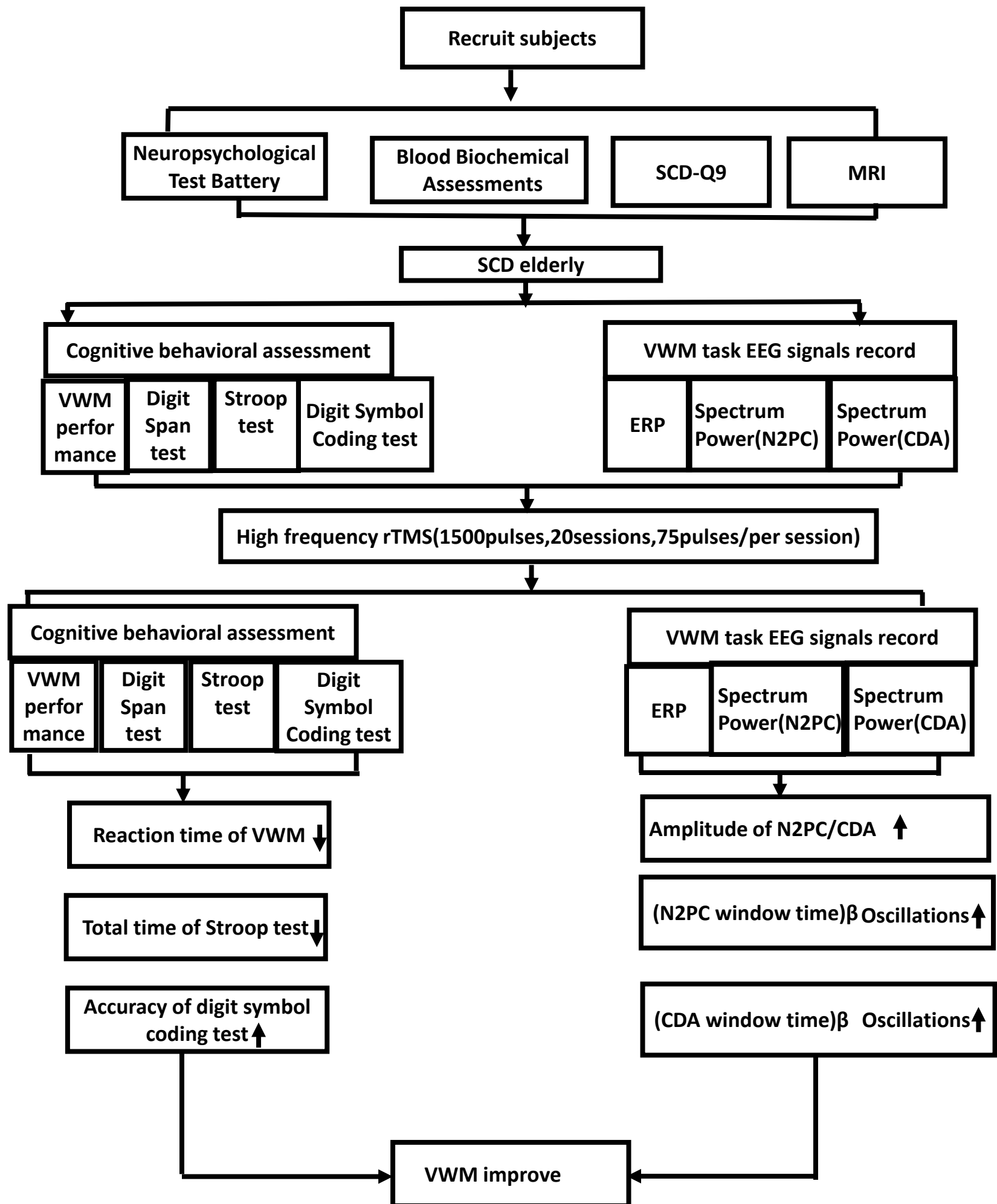

Supplement: Supplementary file 1 [file Data_Sheet_1.PDF]
